# Distal Radius Salter-Harris III Transitional Fracture in an Adolescent Male

**DOI:** 10.1155/2021/5535109

**Published:** 2021-08-03

**Authors:** Adam Kurland, Brian Batko, Jeremy Hreha, Ashley Ignatiuk

**Affiliations:** ^1^Department of Orthopaedic Surgery, Rutgers New Jersey Medical School, New Jersey, USA; ^2^Department of Surgery, Plastic & Reconstructive Surgery Division, Rutgers New Jersey Medical School, New Jersey, USA

## Abstract

In contrast to the well-described Tillaux fracture of the distal tibia, transitional fractures of the distal radius are exceedingly rare and have yet to be well described. Thus far, their presence in the literature has been limited to case reports and a singular series. None have involved a Salter-Harris III fracture pattern. We present the case of a 16-year-old male who sustained a Salter-Harris III transitional fracture of the distal radius with an associated ulnar styloid avulsion fracture secondary to a fall that was treated nonoperatively. Similar to the Tillaux fracture, examination of the distal radius transitional fracture should include computed tomography scan to better illustrate the pattern of injury and guide treatment.

## 1. Introduction

Transitional fractures are intra-articular fractures that occur during the process of physeal fusion in adolescents. It is estimated that up to 20% of physeal fractures in adolescents are transitional fractures [1]. They are most frequently found at the distal tibia [2], and the physiology of the closure of this physis is well understood [3–5]. Fractures of the distal radius are the most common pediatric fracture [6]; however, transitional fractures at this location are rare, and the physiology of the physeal closure of the distal radius has been only minimally elucidated [7]. Only one study has investigated this physeal closure using sequential MRIs and found that mineralization of this physis begins centroradially then ulnarly and progresses in a counterclockwise direction with completion at the peripheral dorsoradial area [7]. Their data also indicate that the process takes less than a year to complete, which suggests why this fracture pattern may be so uncommon [7].

To date, there have been few reports involving transitional fractures at the distal radius. A limited number of case reports and a case series involve triplane fractures of the distal radius but none yet that describe a Salter-Harris III fracture pattern [8–11]. We thus present this case of a 16-year-old male with a transitional, Salter-Harris III fracture of the dorsal ulnar corner of the distal radius.

## 2. Case Report

A 16-year-old right hand dominant Hispanic male presented to the emergency department complaining of left wrist pain after falling off of a bicycle onto an outstretched hand one day prior. On examination, the patient's skin was intact, and he had minor swelling from the midforearm to the hand. He was tender over the distal radius and ulnar styloid. No distal radioulnar joint instability was identified on exam when compared to the contralateral wrist. Motor strength was normal for his hand intrinsic muscles, but wrist flexion and extension were limited secondary to pain. Sensation was intact to light touch over the volar and dorsal aspects of the hand in median, ulnar, and radial nerve distributions. He had a palpable radial pulse and brisk capillary refill at all digits.

**Figure 1:**
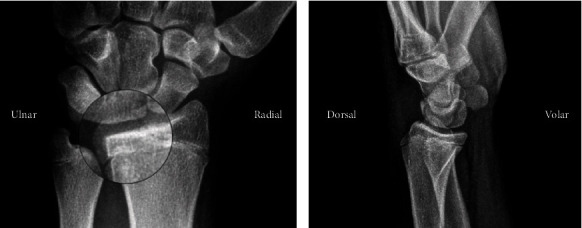
Plain PA and lateral radiographs of the left wrist demonstrating intra-articular fracture at dorsoulnar lip of the radius.

Plain radiographs of the left wrist showed a minimally displaced ulnar styloid avulsion fracture and a Salter-Harris III fracture of the distal radius involving only the dorsal ulnar corner which progressed from the dorsoulnar end of the distal radial epiphysis in the sagittal plane proximally into the physis separating the dorsal and volar ulnar rims and extending ulnarly through the physis and into the distal radial ulnar joint (Figure 1). There was no articular step-off. Also visible was the predominantly closed physeal plate and mild negative ulnar variance. The patient was placed in a well-padded sugar-tong splint and made nonweight bearing to his left upper extremity.

**Figure 2:**
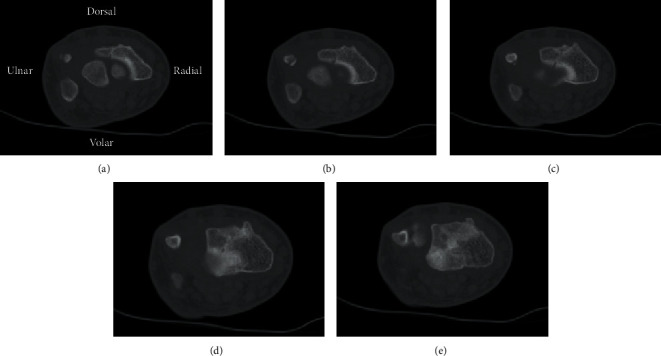
Sequential axial CT views (proximal to distal) demonstrating intra-articular extent of the fracture through the dorsal rim of the radial physis. (d) Shows the radial epiphysis fully in view, revealing the most volar extent of the fracture line dorsal to the volar ulnar rim.

**Figure 3:**
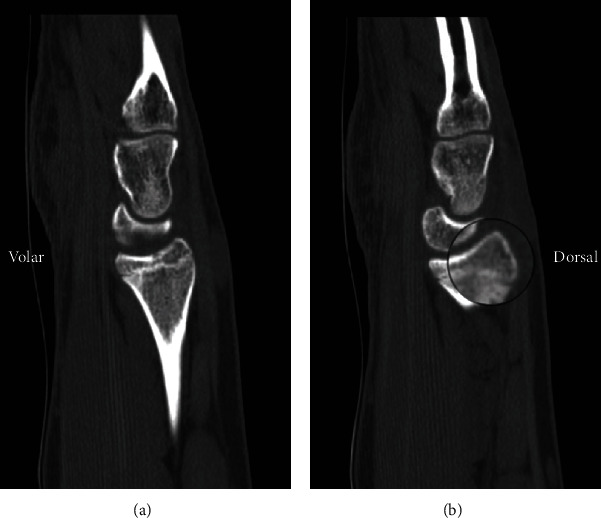
Sagittal CT view showing intra-articular extent of the fracture through the dorsal rim of the radius with interval healing.

**Figure 4:**
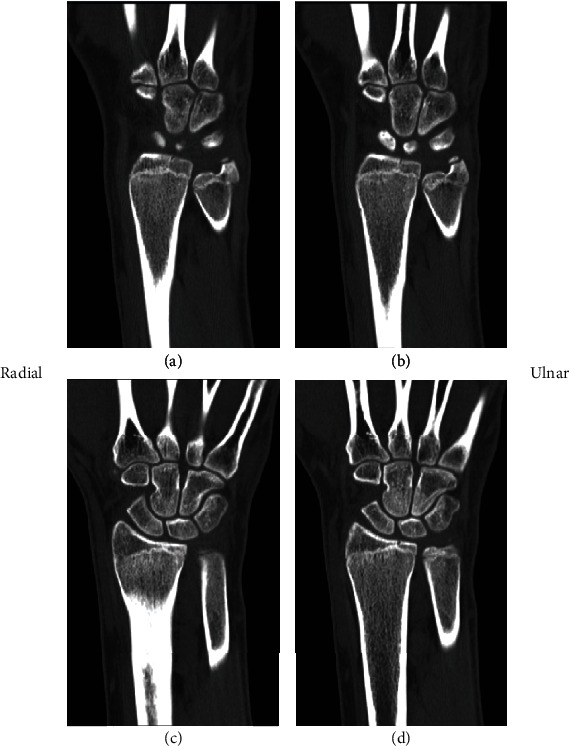
Coronal CT view showing location of fracture at dorsal rim with interval healing and narrowed and fusing radial physis.

The patient was transitioned to a short arm cast at 1.5 weeks with repeat radiographs demonstrating maintained articular alignment and with no distal radioulnar joint instability. A CT scan was attempted at this time but due to patient scheduling conflicts could not be obtained. At 4 weeks after the initial presentation, the cast was removed. The patient had improved range of motion but with only subtle pain and remained tender to palpation at the wrist. CT scan obtained 6 weeks postinjury showed an intra-articular distal radius fracture along the dorsal rim of the radius that had largely healed, while the physis of the distal radius was shown to be narrowed and largely fused **(**Figures 2–4**)**. He was placed in a volar resting splint that could be removed for gentle range of motion exercises. By his latest follow-up visit at 3 months postinjury, the patient had returned to his normal activities of daily living. He was nontender at the wrist and had full wrist flexion and extension, as well as painless radial and ulnar deviation. Due to restrictions from COVID-19, the patient only had a telehealth visit, and thus, no radiographic imaging is available demonstrating a fully healed fracture.

## 3. Discussion

**Figure 5:**
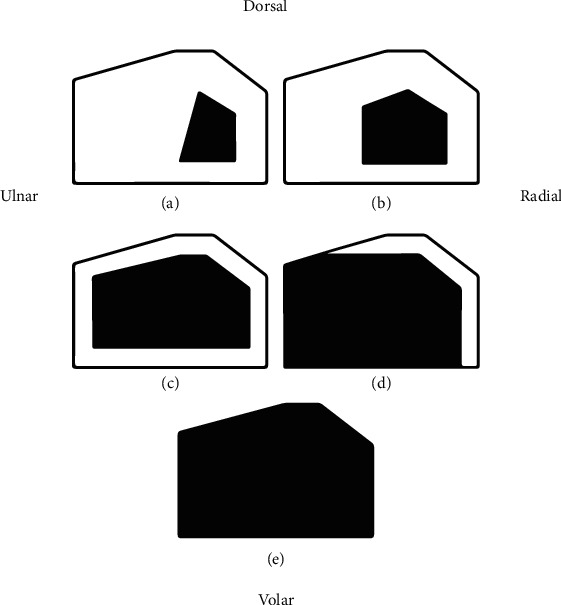
Sequence of fusion of the distal radial physis as identified by Kraus et al. [7]. Reprinted with permission.

In contrast to Tillaux fractures of the distal tibia, transitional fractures of the distal radius are exceedingly rare and have yet to be well described. The physeal closure process of the distal radius takes less than a year to complete [7], as opposed to approximately 18 months in the distal tibia [1], which suggests why this fracture pattern may be so rare. Typically, if the dorsal and volar ulnar rims fragment, they do so concurrently as two distinct pieces. Rarely, one fractures while the other remains intact. The fracture presented in our case involves only the dorsal ulnar corner and traverses the dorsoulnar rim of the patient's physis, which is consistent with the region of the physis that is last to fuse [7] (Figure 5). As such, it is likely the progression of fusion that is responsible for our patient's unusual fracture pattern.

Treatment of transitional fractures of the distal radius should be managed similar to other adolescent transitional fractures. We recommend obtaining a CT scan to better define fracture displacement as this will dictate treatment. Similar to transitional fractures of the distal tibia, we suggest nonoperative management of intra-articular fractures with <2 mm of displacement. If displaced, reduction and screw fixation is recommended. Closed reduction with k-wire fragment manipulation can be attempted; however, if displacement remains, open reduction is necessary. A dorsal approach to the distal radius is recommended to adequately visualize the fracture. Screw fixation across the physis can be performed with minimal deformity risk as patients are near the end of growth and physeal function.

In conclusion, transitional fractures of the distal radius are exceedingly rare and occur due to the physiology of physeal closure described by Kraus et al. [7]. To our knowledge, we are the first to present a case of Salter-Harris III transitional fracture of the distal radius. Treatment of these fractures should be managed similar to other adolescent transitional fractures. Greater than 2 mm of displacement seen on CT scan warrants operative fixation. Further study of Salter-Harris III fractures of the distal radius is needed to elucidate the long-term results of these fractures.

## Data Availability

Data sharing is not applicable to this article as no new data were created or analyzed in this study.
